# *Mycobacterium shimoidei,* a Rare Pulmonary Pathogen, Queensland, Australia

**DOI:** 10.3201/eid2311.170999

**Published:** 2017-11

**Authors:** Timothy M. Baird, Robyn Carter, Geoffrey Eather, Rachel Thomson

**Affiliations:** Princess Alexandra Hospital, Brisbane, Queensland, Australia (T.M. Baird, G. Eather);; Metro South Clinical Tuberculosis Service, Brisbane (T.M. Baird, G. Eather, R. Thomson);; Greenslopes Private Hospital, Brisbane (R. Thomson);; University of Queensland, Brisbane (R. Thomson);; Royal Brisbane and Womens Hospital, Brisbane (R. Carter)

**Keywords:** *Mycobacterium shimoidei*, *M. shimoidei*, nontuberculous mycobacterium, atypical mycobacteria, Queensland, Australia, pulmonary, bacteria, tuberculosis and other mycobacteria

## Abstract

Nontuberculous mycobacteria are human pathogens with increasing incidence and prevalence worldwide. *Mycobacterium shimoidei* is a rare cause of pulmonary disease, with only 15 cases previously reported. This series documents an additional 23 cases of *M. shimoidei* from Queensland, Australia, and highlights the pathogenicity and clinical role of this species.

Nontuberculous mycobacteria (NTM) are prominent human pathogens, with >150 species reported worldwide (*1*). *Mycobacterium shimoidei* is a slow-growing NTM that was first isolated in Japan in 1968, successfully gaining species status in 1975 (*2*). Since then, only 15 cases have been reported worldwide (*3*–*10*).

In Queensland, Australia, NTM is a reportable condition, requiring all isolates to be reported to the Queensland Mycobacterium Reference Laboratory. This series examines all *M. shimoidei* cases in Queensland during January 1, 2000– December 31, 2014.

We extracted data from the Queensland Notifiable Condition System with ethics approval obtained from the Metro North Human Research Ethics Committee (HREC/15/QPCH/65). Confirmatory testing was conducted at the Queensland Mycobacterium Reference Laboratory using 2 methods: the Hain Genotype CM/AS Line Probe Assays (**Hain** Lifescience, Nehren, Germany) and 16S rRNA sequencing.

We obtained clinical information from treating physicians and patient medical records. We recorded each isolate as being likely clinically significant, possibly significant, or unlikely significant, and as being consistent or not with NTM lung disease according to the 2007 American Thoracic Society and Infectious Disease Society of America criteria.

Specimens from 23 patients (35 total isolates) cultured *M. shimoidei* in Queensland during the study period. Individual clinical characteristics, treatment, and outcomes can be seen in the Table. Previously reported cases are summarized in the Technical Appendix.

**Table:** Clinical characteristics, treatment, and outcomes of *Mycobacterium shimoidei* isolates, Queensland, Australia*

Specimen (isolates)	Age, y/sex	Significant	Signs/ symptoms	Radiology	Concurrent conditions	Management (time)	Outcome
Sp and Br (×4)	60/M	Likely	C, Sp, WL	Cavities, nodule	COPD, asthma	Observed	Stable
LTis (×1)	56/M	Likely	Died	Unknown	Unknown	None	Died
Sp (×1)	75/F	Likely	C, Sp, WL	Cavities, nodules	COPD, HF, AF, GERD	None	Died of other cause
Sp (×3)	72/M	Likely	C, D, WL	Cavity, nodules	COPD, bronchiectasis, IHD	Observed	Died of lung disease
LTis (×1)	62/F	Likely	C, WL, NS	Cavity	None	INH, RFP, PZA, EMB (6 mo)	Stable
Sp (×2)	68/M	Likely	C, Sp, H, WL, Fa	Cavities, consolidation	COPD, aspergillus, HTN	CLA, MFX, SMX (12 mo)	Improved
Sp and Br (×4)	70/M	Likely	C, Sp, CP	Cavities	Lung cancer, COPD, bronchiectasis	CLA, RIF, EMB (12 mo)	Died of lung disease
LTis (×1)	77/F	Likely	C, WL, Fa	Cavity, nodules	COPD, GERD	CLA, RFP, EMB (18 mo)	Improved
Sp (×3)	68/M	Likely	C, Sp, WL	Cavity, consolidation	COPD, RA, anemia	Observed	Stable
Br (×1)	76/M	Possibly	D, WL	Nodules	COPD, anemia	None	Unknown
Br (×1)	84/M	Possibly	C, Sp	Mass, effusion	Lung cancer, GERD	Observed	Died of lung disease
Sp (×1)	84/M	Possibly	C, D, Fa	Consolidation	COPD, bronchiectasis	Observed	Improved
Sp (×1)	29/M	Possibly	C, D, WL	Nodules	CF, bronchiectasis	AMK, CFX, AZA, CFZ (24 mo)	Improved
Sp (×1)	74/F	Possibly	C, Sp	Nodules, consolidation	Bronchiectasis	Observed	Improved
Sp (×5)	84/F	Possibly	C, Sp, H, WL	Nodules	Bronchiectasis, type 2 diabetes, HTN	CLA (2 mo)	Improved
Sp (×1)	58/M	Possibly	C, Sp	Normal	Obesity, HTN	Observed	Stable
Sp (×1)	57/M	Unlikely	Unknown	Unknown	Unknown	Unknown	Unknown
LTis (×1)	55/F	Unlikely	Unknown	Unknown	Unknown	Unknown	Unknown
Sp (×1)	67/M	Unlikely	Unknown	Unknown	Unknown	Unknown	Unknown
Sp (×1)	60/M	Unlikely	C, D	Normal	Asthma	None	Unknown
Sp (×1)	59/F	Unlikely	C	Normal	Asthma, GERD	None	Unknown
Sp (×1)	73/M	Unlikely	Unknown	Unknown	Unknown	Unknown	Unknown
Sp (×1)	54/M	Unlikely	Unknown	Unknown	Unknown	Unknown	Unknown

*AF, atrial fibrillation; AMK, amikacin; AZA, azithromycin; Br, bronchoscopic washing; C, cough; CF, cystic fibrosis; CFX, cefoxitin; CFZ, clofazimine; CLA, clarithromycin; COPD, chronic obstructive pulmonary disease; CP, chest pain; D, dyspnea; EMB, ethambutol; Fa, fatigue; GERD, gastroesophageal reflux disease; H, hemoptysis; HF, heart failure; HTN, hypertension; IHD, ischemic heart disease; INH, isoniazid; LTis, lung tissue; MFX, moxifloxacin; NS, night sweats; PZA, pyrazinamide; RA, rheumatoid arthritis; RFP, rifampin; RIF, rifabutin; SMX, sulfamethoxazole, Sp, sputum; WL, weight loss.

Sixteen (69.6%) patients were male (mean ± SD age 66.2 ± 12.6 years), consistent with previous case reports (*3*–*10*). Nine (39.1%) were classified as being likely clinically significant and 7 (30.4%) possibly significant. Ten patients (43.5%) met the 2007 American Thoracic Society and Infectious Disease Society of America criteria for having NTM lung disease. All isolates were cultured from respiratory specimens, with 15 (65.2%) isolated from sputum; 2 (8.7%) from bronchial washings; 2 (8.7%) from both bronchial washings and sputum; and 2 (17.4%) from lung tissue either with computed tomography–guided biopsy or at autopsy. Only 4 (17.4%) specimens were smear-positive by microscopy.

The most common symptoms were cough or sputum (16; 69.6%); weight loss (9; 39.1%); dyspnea (5; 21.7%); fevers or sweats (4; 17.4%); and fatigue (2; 8.7%). Cough and sputum predominated in previous cases, but not weight loss (*3*–*10*). Radiology demonstrated cavitary disease in 9 patients (39.1%). Similar to our cohort, 9 of the 15 previously reported cases had cavities, highlighting a potentially distinguishing feature of *M. shimoidei* lung disease (*3*–*7*).

The most common associated concurrent conditions were obstructive airway disease (10; 43.5%), bronchiectasis (6; 26.1%), gastroesophageal reflux disease (4; 17.4%), and malnutrition (3; 13.0%). Underlying chronic lung disease was also present in previously reported cases and included chronic obstructive pulmonary disease, past tuberculosis, pneumoconiosis, and bronchiectasis (*3*–*10*).

Although 16 patients (69.5%) were deemed to have either likely or possibly clinically significant disease, only 6 (26.0%) underwent medical treatment, with 7 (30.4%) being actively observed. These low treatment numbers may reflect a lack of knowledge in relation to *M. shimoidei*; however, they may also be an indirect result of the underlying comorbidities and poor functional status of infected patients.

When medical treatment was offered, however, 5 of the 6 patients improved or had stable disease, with the sixth patient dying of lung cancer while undergoing antimicrobial therapy. Of the 7 patients who were observed, 3 remained stable, 2 improved, and 2 died of either chronic lung disease or progression of their *M. shimoidei* infection. In comparison, 6 of the 15 previous cases in the literature improved with medical treatment, with 4 dying during treatment and 1 remaining stable with observation alone (*3*–*10*). Although this relatively high death rate may reflect the nature of the patients’ comorbidities, it still highlights the clinical significance of *M. shimoidei* if isolated.

Although none of the Queensland cohort underwent drug susceptibility testing, review of previous cases suggests that a combination of rifabutin, ethambutol, and clarithromycin may be an effective drug regimen, with moxifloxacin/levofloxacin, sulfamethoxazole, pyrazinamide, and linezolid as other potential agents (*3*–*7*).

Our study has several limitations. First, it is a retrospective case series with data extracted from a passive surveillance system. Even though all laboratory-confirmed cases were captured, it is possible that not all patients with *M. shimoidei* infection received this diagnosis or were able to provide an appropriate specimen for identification. Futhermore, due to both the clinical characteristics being reported by various treating physicians and a large proportion not having complete clinical or follow-up data available, we may have captured inaccurate or inconsistent data.

This case series highlights the clinical significance and pathogenicity of *M. shimoidei.* Cases have been isolated only from respiratory specimens, occur predominantly in male patients with underlying chronic lung disease, and commonly present with cavitary disease. Although illness and death are associated with *M. shimoidei* infection, a reasonable outcome can be achieved with treatment. Possible drug regimens involve a combination of rifabutin, ethambutol, and clarithromycin, with moxifloxacin/levofloxacin, sulfamethoxazole, pyrazinamide, and clofazimine also potentially being useful. Increased recognition and understanding of this pathogenic organism are necessary to improve patient outcomes.

Technical Appendix Clinical characteristics, treatment, and outcomes of previously reported *Mycobacterium shimodei* isolates, Queensland, Australia.
