# Supplementary material for: Mycobacterium shimoidei, a Rare Pulmonary Pathogen, Queensland, Australia
Source: Emerg Infect Dis. 2017 Nov;23(11):1919–22. doi: 10.3201/eid2311.170999 (PMC5652447; doi:10.3201/eid2311.170999)
Supplement: Technical Appendix — Clinical characteristics, treatment, and outcomes of previously reported Mycobacterium shimodei isolates, Queensland, Australia. [file 17-0999-Techapp-s1.pdf]

Article DOI: https://doi.org/10.3201/eid2311.170999

Mycobacterium shimoidei, a Rare Pulmonary Pathogen, Queensland, Australia

Technical Appendix Table. Clinical characteristics, treatment, and outcomes of previously reported *Mycobacterium shimodei* isolates*

Author, year	Country	Age, y, sex	Symptoms	Radiology	Comorbidities	Management	Outcome
Tsukumura et al., 1975 (*1*)	Japan	56/M	Unknown	Cavity	Unknown	Unknown	Died of lung disease
Rusch-Gerdes et al., 1985 (*2*)	Germany	77/M	F	Cavity	Silicosis	INH, PRO, RFP, SM, ISO (3 mo)	Improved
Chromyc et al., 1989 (*3*)	Canada	84/M	C, S	Consolidation	COPD, pneumoconiosis	Unknown	Died of lung disease
Tortoli and Simonetti, 1989 (*4*)	Italy	68/M	U	Cavity	Old TB, Addison's	INH, SM, EB, RFP, KM, PAS (4 mo)	Died of lung disease
Miller et al., 1991 (*5*)	Canada	65/M	F	Mass	COPD, lung Ca	Unknown	Unknown
Furrer,et al.,1994 (*6*)	Switzerland	34/M	U	Unknown	HIV	None	Died of other cause
Heller et al., 1996 (*7*)	France	48/F	A	Cavity, nodules	Old TB	SM, EMB, CLA, RIF; EM, CPFX, RIF (18 mo)	Improved
Auregan et al., 1997 (*8*)	Madagascar	43/F	H	Cavity	Old TB	INH, RFP, PZA, EMB, SM (17 mo)	Died of lung disease
Goudge et al., 1998 (*9*)	Australia	75/M	C, S, WL, NS	Cavity, nodules	COPD, previous lobectomy	EMB, PZA, CLA, RIF (5 wk)	Died of lung disease
Mayall et al., 1999 (*10*)	Australia	53/F	C, S, WL	Cavity	COPD, fibrosis, esophageal Ca	INH, RFP, PZA, EMB (10 d)	Died of lung disease
Koukila-Kahkola et al., 2000 (*11*)	Finland	78/F	C, S	Normal	None	Observation	Stable
Sundman et al., 2000 (*12*)	Sweden	59/F	F, CP, D	Cavity	COPD	CPFX, CLDM; MDZ, TFX (6 wk)	Improved
Takayama et al., 2005 (*13*)	Japan	68/M	F, C	Consolidation	COPD	RFP, EMB, CAM, PZA, CPFX (6 mo)	Improved
Kanaji et al., 2011 (*14*)	Japan	83/M	C, S	Cavity	COPD	CLA, RFP, EMB (18 mo)	Improved
Galizzi et al., 2013 (*15*)	Italy	53/F	C, H	Nodules	Bronchiectasis	AMK, RIF, EMB, CLA (18 mo)	Improved

* A, asymptomatic; AMK, amikacin; C, cough; CLA, clarithromycin; CLDM, clindamycin; COPD, chronic obstructive pulmonary disease; CPFX, ciprofloxacin; D, dyspnea; EMB, ethambutol; F, fever; H, hemoptysis; INH, isoniazid; KM, kanamycin; MDZ, metronidazole; NS, night sweats; PAS, para-aminosalicylic acid; PRO, prothionamide; PZA, pyrazinamide; RFP, rifampin; RIF, rifabutin; SM, streptomycin; Sp, sputum; TB, tuberculosis; TFX, trifolitoxin; U, unknown; WL, weight loss

References

1. Tsukamura M, Shimoide H, Shaefer WB. A possible new pathogen of group III *Mycobacteria*. J Gen Microbiol. 1975;88:377–80. http://dx.doi.org/10.1099/00221287-88-2-377

2. Rusch-Gerdes S, Wandelt-Freerksen E, Schroder K-H. Vorkommen von *Mycobacterium shimoidei* in der Bundes-republick Deutschland. Zentralb Bakteriol Hyg Orig Reihe A. 1985;259:146–50.

3. Chomyc SA, Pearson JH, Helbecque D. *Mycobacterium shimoidei*—Alberta. Can Dis Wkly Rep. 1990;17:85–6.

4. Tortoli E, Simonetti MT. Isolation of *Mycobacterium shimoidei* from a patient with cavitary pulmonary disease. J Clin Microbiol. 1991;29:1754–6.

5. Miller MA, Eymard D, Thibert L. *Mycobacterium shimoidei*: first reported isolate in Canada. Can Dis Wkly Rep. 1991;17:11–2.

6. Furrer H, Bodmer T, von Overbeck J. Disseminated nontuberculous mycobacteriosis in AIDS patients. Schweiz Med Wochenschr. 1994;124:96–8.

7. Heller R, Jaulhac B, Charles P, De Briel D, Vincent V, Bohner C, et al. Identification of *Mycobacterium shimoidei* in a tuberculosis-like cavity by 16S ribosomal DNA direct sequencing. Eur J Clin Microbiol Infect Dis. 1996;15:172–5. http://dx.doi.org/10.1007/BF01591494

8. Auregan G, Ramaroson F, Génin C, Vincent Lévy-Frébault V. Un cas d’infection pulmonaire à *Mycobacterium shimoïdei* à Madagascar. Bull Soc Pathol Exot. 1997;90:75–7.

9. Goudge RJ, Mayall BC, Leslie DE, Holmes PW, Robinson SL. An Australian isolate of *Mycobacterium shimoidei*. Pathology. 1998;30:399–401. http://dx.doi.org/10.1080/00313029800169706

10. Mayall B, Gurtler V, Irving L, Marzec A, Leslie D. Identification of *Mycobacterium shimoidei* by molecular techniques: case report and summary of the literature. Int J Tuberc Lung Dis. 1999;3:169–73 http://www.ncbi.nlm.nih.gov/pubmed/10091886.

11. Koukila-Kähkölä P, Paulin L, Brander E, Jantzen E, Eho-Remes M, Katila ML. Characterisation of a new isolate of *Mycobacterium shimoidei* from Finland. J Med Microbiol. 2000;49:937–40. http://dx.doi.org/10.1099/0022-1317-49-10-937

12. Sundman K, Chryssanthou E, Petrini B. *Mycobacterium shimoidei*, an easily misdiagnosed non-tuberculous pulmonary mycobacterium. Scand J Infect Dis. 2000;32:450–1 http://www.ncbi.nlm.nih.gov/pubmed/17338311.

13. Takayama S, Tominaga S, Tsukada Y, Ohkochi M, Inase N. [A case of pulmonary *Mycobacterium shimoidei* infection]. Kekkaku. 2006;81:537–41.

14. Kanaji N, Kushida Y, Bandoh S, Ishii T, Haba R, Tadokoro A, et al. Membranous glomerulonephritis associated with *Mycobacterium shimoidei* pulmonary infection. Am J Case Rep. 2013;14:543–7. http://dx.doi.org/10.12659/AJCR.889684

15. Galizzi N, Tortoli E, Gori A, Morini F, Lapadula G. A case of mild pulmonary disease due to *Mycobacterium shimoidei* with a favorable outcome. J Clin Microbiol. 2013;51:3467–8. http://dx.doi.org/10.1128/JCM.01028-13
